# A Novel Voltammetric Point-of-Care Device for Rapid and Accurate Nucleic Acid and Viral Pathogen Detection

**DOI:** 10.1021/acsomega.5c12087

**Published:** 2026-06-01

**Authors:** Roberto Munita, Roman Lyttleton, Emelie Danefur, Sviataslau Sasinovich, Karin Wehlin, Tautgirdas Ruzgas, Patrik Medstrand, Jae Yen Shin, Andreas Nyberg, Kushagr Punyani

**Affiliations:** † Diagonal Bio AB, The Spark Medicon Village, Scheeletorget 1, Lund 22381, Sweden; ‡ Biomedical Science, Faculty of Health and Society, Malmö University, Malmö 20506, Sweden; § Biofilms Research Centre for Biointerfaces, Malmö University, Malmö 20506, Sweden; ∥ Clinical Virology, Department of Translational Medicine, Lund University, Lund 22100, Sweden; ⊥ Department of Biochemistry and Molecular Biology, Facultad de Ciencias Químicas y Farmacéuticas, Universidad de Chile, Santiago 8380494, Chile; # Advanced Center for Chronic Diseases, Facultad de Ciencias Químicas y Farmacéuticas, Universidad de Chile, Santiago 1025000, Chile; ∇ Nested Bio AB, Nannas Gata 10, Malmö 21535, Sweden; ○ Nyberg Exploration AB, Regementsgatan 33, Malmö 21753, Sweden

## Abstract

With zoonotic outbreaks on the rise, rapid and accurate infectious disease diagnostics are critical for both human and animal health. Traditional lateral flow assays lack sensitivity and specificity, while isothermal amplification methods like LAMP, though rapid, can be hindered by optical detection issues. We present an electrochemical nucleic acid amplification test (NAAT) that combines isothermal amplification with real-time voltammetric detection. This novel method relies on monitoring of amplification-associated proton release through redox probing of a pH-sensing compound. The method shows high concordance with RT-qPCR under the tested conditions and demonstrates a limit of detection (LOD) of 50 copies/reaction for IAV. In RNA purified from clinical samples, 231/234 (98.7%) are concordant with RT-qPCR for SARS-CoV-2, IAV, IBV, or RSV A. In a proof-of-concept extraction-free workflow, 14/14 equine nasal swab samples are correctly classified for EHV-5 relative to qPCR. Together, these data support the feasibility of real-time voltammetric LAMP across several sample types, while broader point-of-care validation across additional matrices and prospective cohorts remains to be established.

## Graphical abstract

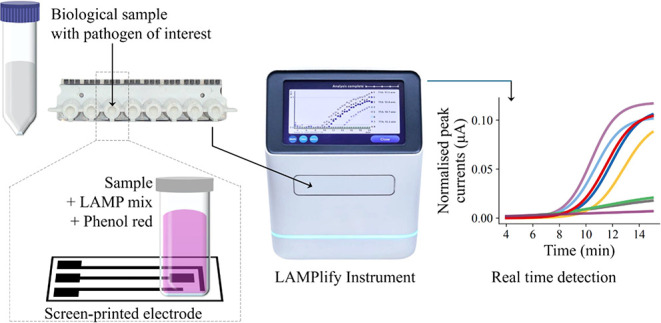

## Introduction

With several zoonotic outbreaks over the last century, as infectious diseases have transcended species and geographic boundaries, the need to advocate for unified One Health approaches to tackle infectious diseases between humans and animals has become evident.^1,2^ This also underlines the critical need for rapid, accurate and affordable pathogen detection that can be deployed for infectious disease diagnostics for both human and veterinary use, surveillance as well as disease management response to prevent widespread outbreaks. While centralized diagnostics rely on technologies such as quantitative polymerase chain reaction (qPCR) and protein detection methods like enzyme-linked immunosorbent assay (ELISA), they are limited by long turnaround times, the need for specialized equipment and expertise, and labor-intensive extraction procedures. On the other hand, traditional point-of-care diagnostics like lateral flow assays provide accessibility and simplicity but often fall short on sensitivity and specificity.^3^

The inability to rapidly and accurately detect pathogens in the field has prompted exploration of alternative pathogen detection methodologies, with Isothermal Amplification (ISA) emerging as a promising solution. This includes techniques such as Rolling Circle Amplification (RCA) and Loop-mediated Isothermal Amplification (LAMP), that eliminate the need for complex thermal cycling by utilizing specialized enzymes that facilitate nucleic acid amplification at a constant temperature and have also been reported to accommodate complex sample types.^4^ However, these assays can be hampered by bulk and optical characteristics of the sample such as the turbidity produced during amplification impeding direct monitoring of reaction progress,^5^ or complex samples interfering with colorimetric detection as the reporting mechanism, thereby requiring nucleic acid extraction and/or purification anyway.^6,7^

Electrochemical detection presents a convenient alternative reporting mechanism as it facilitates surface detection instead of bulk sample probing, thereby addressing some of the limitations of optical detection. Electrochemical detection has been reported in combination with ISA using a variety of methodologies. First, this can be achieved by the addition of reporter molecules that react with amplification byproducts to yield a measurable reaction or product, thereby facilitating end-point analysis,^8^ but largely precluding real-time detection. Alternatively, reaction progress or end-point measurements can be enabled by exploiting detection of the amplified DNA by its interactions with electroactive intercalating or binding moieties such as methylene blue, either through solution-phase detection^9–11^ or a capture probe-mediated bead-based or surface-based capture.^12^ However, these approaches often rely on DNA-binding intercalators that can interfere with the ISA process and may only facilitate end-point detection^8–10,13^ or lead to reduced sensitivity.^11^ To circumvent these issues, we devised an electrochemical assay using phenol red, a nonintercalating pH indicator. Unlike DNA-binding dyes, phenol red in optimized commercial formulations has been shown to yield amplification performance comparable to standard, dye-free LAMP chemistries,^14^ thereby enabling real-time detection without inhibiting the reaction. Compared with end-point phenol-red colorimetric LAMP, the real-time voltammetric readout enables objective algorithmic calling from a sigmoidal kinetic signature (TTA) within a fixed window. This reduces susceptibility to subjective interpretation and to late nonspecific pH shifts (e.g., primer-derived artifacts) that can occur after prolonged incubation.

During NAAT or ISA, as nucleic acid amplification progresses, protons are released. Specifically, LAMP can tolerate an unbuffered or lightly buffered reaction mix, which allows for a drop in the pH of the solution^15^ during amplification. We leveraged the electrochemical properties of pH-sensing compound phenol red to monitor ISA in real-time using electrochemistry. Unlike prior electrochemical LAMP approaches relying on phenol red-mediated detection^16,17^ that have only demonstrated end-point analysis, we exploited the continuous pH decrease during LAMP to develop a real-time, highly accurate, and rapid NAAT assay. By continuously monitoring the pH changes through voltammetry, we demonstrate precise real-time measurement of amplification progress in lightly buffered LAMP reactions. Unlike end-point colorimetric methods, which are susceptible to false positives from nonspecific background acidification caused by primer-dimer formation or late-stage self-priming events,^18,19^ real-time voltammetric monitoring enables three key advantages: (i) kinetic discrimination of true positives from baseline drift via sigmoidal curve analysis, (ii) faster clinical decision-making through early detection of high-viral-load samples without waiting for full incubation, and (iii) semiquantitative viral load stratification based on time-to-amplification (TTA). Employing phenol red as the key electroactive redox reporter in our voltammetric readout: its redox peak shifts with pH (≈65 mV/pH), allowing the pH drop generated during LAMP amplification to be transduced into a measurable electrochemical signal.

Here, this voltammetric monitoring strategy is implemented in the integrated LAMPlify device for real-time detection of several pathogens in clinical, veterinary, and microbiological samples. The present study evaluates its performance under the tested conditions and its potential for future point-of-care deployment.

## Experimental Section

### pH Response of Phenol Red

A Palmsens EmStat3 Blue potentiostat is connected to a DBLC-8000 screen-printed electrode held at a controlled temperature by a VWR ADV-2 Digital Heatblock. A set of corresponding solutions ranging in pH from 8.4 to 5.5 were created by titrating an 8 mM tris buffer, 0.2 mM phenol red solution with HCl/NaOH respectively. Square wave voltammetry (frequency 10 Hz, amplitude 20 mV, step size 5 mV, range −0.1 V to −0.9 V) was applied to characterize the current response of 1 mL aliquots of these solutions. The screen-printed electrodes were used without any cleaning or surface preparation performed prior to measurements. This approach was maintained throughout all subsequent experiments to reflect the intended point-of-care use case, where electrode pretreatment would not be practical.

### Targets, Primers, Reaction Master Mix

Synthetic targets were purchased from Twist Bioscience (Twist Synthetic SARS-CoV-2 RNA Control ref MN908947.3, Twist Synthetic Influenza H1N1 (2009) RNA Control ref 103016) and ATCC (ATCC Quantitative Genomic RNA from Influenza B virus (Yamagata Lineage) strain B/Wisconsin/1/2010 BX-41A) while chemically inactivated viral samples were obtained from Zeptometrix (NATtrol Respiratory Verification Panel NATRVP-NNS). γ-irradiated inactivated SARS-CoV-2 samples were obtained from BEI Resources (NR-52287). All primers were purchased from Integrated DNA Technologies, in standard desalting form. The LAMP Mastermix M1800 WarmStart Colorimetric LAMP Master Mix was purchased from New England Biolabs. Diagonal Bio Lysis buffer was designed as a low buffer capacity liquid to collect material from nasal swabs and prepared to contain 3 mM Tris-HCl (ThermoFisher), 500 mM Guanidinium Sulfate (GuSO_4_) (Sigma-Aldrich), 1 mM EDTA (Invitrogen), and titrated to pH 8.

### LAMP Reactions

All LAMP reactions were performed at 65 °C with the following primer concentrations: Forward inner primer (FIP) and Backward inner primer (BIP) at 1.6 μM, Forward outer primer (F3) and Backward outer primer (B3) at 0.2 μM, Loop forward primer (LF) and Loop backward primer (LB) at 0.4 μM. Unless otherwise specified, the final reaction volumes were 50 μL and contained 5 μL of sample. A complete list of LAMP primers used is provided in Supporting Information Table S1.

### RT-qPCR

RNA standards for SARS-CoV-2 were obtained from the American Type Culture Collection (ATCC; VR-3276SD). Standards for Influenza A and Influenza B were sourced from VirCell as AMPLIRUN Influenza A H1 RNA control (MBC028) and AMPLIRUN Influenza B RNA control (MBC030), respectively. An additional standard for Respiratory Syncytial Virus (RSV) subtype A was obtained from VirCell as AMPLIRUN Respiratory Syncytial Virus (subtype A) RNA control (MBC041). Quantitative real-time polymerase chain reaction (qPCR) for SARS-CoV-2, Influenza A, and Influenza B was conducted using the qPCRBIO Probe 1-Step Virus Detect Kit (PCR Biosystems) on a CFX96 Real-Time PCR Detection System (Bio-Rad). Primers and probes specific to these targets were synthesized by Integrated DNA Technologies and employed in accordance with the CDC Influenza SARS-CoV-2 Multiplex Assay protocol.^20^ A complete list of RT-qPCR primers used is provided in Supporting Table S2. Reaction mixtures were prepared following the manufacturer’s instructions for the qPCRBIO Probe 1-Step Virus Detect Kit. Amplification was performed with the following thermal cycling parameters: reverse transcription at 25 °C for 2 min, followed by 50 °C for 15 min; initial denaturation at 95 °C for 3 min; and 45 cycles of denaturation at 95 °C for 15 s and annealing-extension at 55 °C for 30 s. Fluorescence data were acquired during the annealing-extension phase at 55 °C.

For RSV detection, RT-qPCR was performed separately using the qPCRBIO Probe 1-Step Virus Detect Kit (PCR Biosystems) on the same CFX96 Real-Time PCR Detection System (Bio-Rad). Primers and probes targeting RSV subtypes A and B were designed as described by Todd et al.^21^ Reaction mixtures were prepared according to the manufacturer’s instructions, and amplification was conducted with the following conditions: reverse transcription at 45 °C for 10 min; initial denaturation at 95 °C for 3 min; and 40 cycles of denaturation at 95 °C for 15 s and annealing-extension at 60 °C for 30 s. Fluorescence data were collected during the annealing-extension phase at 60 °C.

### Clinical and Veterinary Samples

Clinical specimens, obtained via nasopharyngeal swabs and confirmed positive for respiratory viral infections, were sourced from the regional biobank of Southern Sweden (Region Skåne, Lund) between 2017 and 2020. To ensure anonymity, all clinical samples were blinded, and only information regarding the infecting pathogen was provided to the technical laboratory staff. Ethical approval for this study was granted by the Swedish Ethical Review Authority (Etikprövningsmyndigheten), Stockholm, Sweden (Approval No. 2020–02781).

These nasopharyngeal swab clinical specimens had RNA extracted using a standard magnetic-bead workflow (MagaBio Plus Virus RNA Purification Kit II on the GenePure Pro system) and were analyzed with RT-qPCR both at time of acquisition as well as during this study. Samples which had a discrepancy between these RT-qPCR analyses were discarded and not included in this study.

For EHV-5 detection, samples were extracted by adding the Diagonal Bio Lysis buffer directly to the Zymo Research Quick-DNA Microprep kit in place of the supplied buffer and otherwise following the manufacturer’s protocol. qPCR was performed on a CFX96 Real-Time PCR Detection System (Bio-Rad) using SsoAdvanced Universal SYBR Green Supermix (Bio-Rad). All primers were synthesized by Integrated DNA Technologies. The qPCR protocol was as follows: initial denaturation at 98 °C for 3 min; and 40 cycles of denaturation at 98 °C for 10 s and annealing-extension at 60 °C for 10 s. Fluorescence data were acquired during the annealing-extension phase at 60 °C.

### Limit of Detection (LOD) Studies

Serial dilutions of Influenza A H1N1 RNA standards were prepared in TE buffer. The LOD was defined as the lowest concentration at which ≥95% of replicates were positive. An initial screening was performed with 4 replicates ranging from 0 to 10000 copies/reaction, followed by a confirmatory study of 20 replicates at the provisional LOD concentration.

### Logistic Curve Fitting and TTA Classification

Reaction progress was estimated by either the peak position (V) or the current differential (μA) at two specific potentials. These were fit with an asymmetric 5-parameter logistic curve^22^ by minimizing the square of the residuals using the python package scipy. For the point-of-care analysis, this 5-parameter sigmoidal fitting is implemented in the embedded processing of the portable device using a custom implementation of a gradient-descent nonlinear regression algorithm. This processing happens in real time with minimal computational burden. Time to amplification (TTA) was calculated as the point at which this curve exceeded a specific threshold (0.025 μA unless otherwise specified). To distinguish between amplification and other nonspecific drift, samples were only considered positive if they exceeded the aforementioned threshold and contained a gradient greater than 0.005 μA/min.

## Results and Discussion

### pH Dependence of Biosensor Response

A set of phenol red current responses was obtained for solutions with varying pH. These spanned a pH range from 8.4 to 5.5. Square wave voltammetry was performed and a specific current response extracted by sequentially collecting 5 identical sweeps taken at 30 s intervals and plotting the average of the last 3 sweeps as shown in Figure 1A. The first two sweeps were discarded due to signal fluctuation attributed to electrode/solution interface stabilization. The minimum current of each trace was considered the “peak” and plotted against pH (see Figure 1B) that revealed higher peak potentials at lower pH. The change of potential vs pH was estimated using a weighted linear regression as 65 ± 4 mV/pH which is in good agreement with the expected two-electron, two-proton redox reaction and 67 mV/pH expected^23^ for phenols at 65 °C.

**Figure 1:**
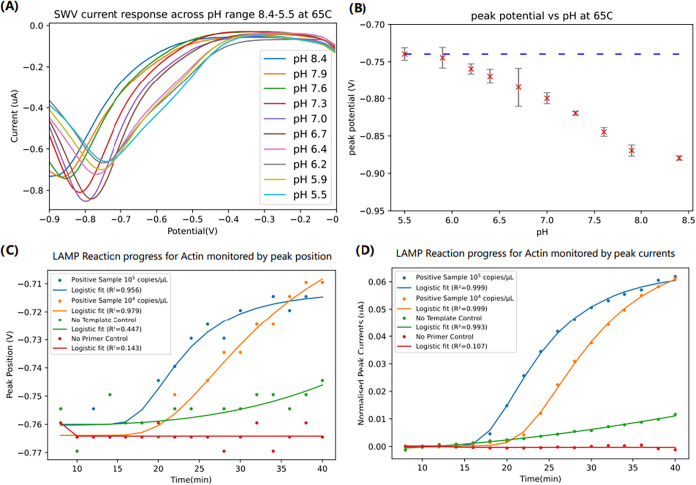
Real-time electrochemical monitoring of LAMP amplification via square-wave voltammetry of the pH-responsive redox reporter phenol red. (A) SWV current response of phenol red from pH 8.4 to 5.5 at 65 °C, (B) SWV peak potential of phenol red (V) vs pH at 65 °C. Reaction progress of LAMP reactions containing actin and corresponding primers (105 copies, 104 copies, no-template control, no-primer control respectively) over time monitored by (C) peak position­(V), (D) normalized peak currents (μA).

### Electrochemically Monitored LAMP Reaction

To engineer a positive amplification LAMP reaction, an actin template was added to LAMP Mastermix containing a corresponding actin specific primer set. The complete current–voltage traces (I–V curves) at all time points can be seen in Supporting Figure S1 for 10^5^ copies and 10^4^ copies of actin as well as no-template and no-primer controls. Figure 1C plots the peak position of each of these traces for the entire duration of the LAMP reaction. As the LAMP reaction progresses, peak width reduces and the peak potential increases, changing sharply after an initial lag phase, characteristic of many nucleic acid amplification reactions.^24^ Conversely, neither negative control showed any significant change in the peak.^15^

Individual I–V curves can exhibit variability due to electrical measurement noise, electrode drift, and/or surface/interface effects. As a result, direct peak-position tracking shows noticeable scatter (Figure 1C). To obtain a more robust reaction-progress metric, we use a two-step normalization: (i) each voltammogram is internally referenced by computing the current differential at two fixed potentials (Δ*I*), reducing sensitivity to peak-picking noise and global drift; and (ii) a baseline estimated during the early lag phase is subtracted to correct for starting-point offsets. This processing substantially reduces noise and yields a stable sigmoidal trace suitable for defining time-to-amplification (TTA) (Figure 1D). For computational simplicity and ease of implementation on an integrated circuit (IC), this normalization was achieved by calculating the difference between current values recorded at two fixed potentials: −0.73 V and −0.85 V.

The potential at −0.73 V was selected as a reference because it corresponded to the saturation value observed in Figure 1B, the point at which further reductions in solution pH no longer shifted the peak potential. Due to measurement constraints, saturation at high pH could not be observed, as the potential range was limited to −0.9 V to minimize unintended electrolysis. The second potential, −0.85 V, was chosen near the upper limit of the measurement range to avoid edge effects while still allowing for stable signal acquisition. A parameter optimization analysis through a heat map was also performed to systematically assess and support the choice of subtraction potentials (Supporting Figure S3). Furthermore, each current value was computed as a 5-point moving average centered on the desired potential, providing a further reduction in noise. To ensure a stable measurement of reaction progress, the average difference between the two current values was calculated during the lag phase (3–7 min) and used as a baseline. This baseline was then subtracted from all time points, providing a drift-corrected signal for analysis.

This baseline subtraction corrects for small baseline offsets arising from modest variation in the initial pH of the input samples. While this was sufficient for the sample types evaluated in this study, input samples with extreme initial pH (either unusually acidic or alkaline), particularly if they are also highly buffered (e.g., standard UTM/VTM), could affect assay performance. Further work will be required to define a broadly applicable safe operating input pH range across diverse sample matrices.

Five parameter sigmoidal fits were chosen as they are a good model for these kinds of nucleic amplification reactions^25^ and the regression coefficients (*R*2) computed. The higher *R* ^2^ 0.999 vs 0.956 (10^5^ copies) and 0.999 vs 0.979 (10^4^ copies) for the fits of normalized peak currents compared to peak position (Figure 1C,D) supports the use of this method as a better avenue for real-time monitoring of reaction progress than peak position. This is further supported by the close resemblance of these sigmoidal response curves to typical LAMP amplification kinetics obtained through fluorescence-based detection, presented in Supporting Figure S2.

### Point-of-Care Diagnostic Setup

To enable point-of-care diagnostics, LAMPlify – a compact benchtop, portable instrument was created with integrated microcontrollers, potentiostat, heat source and a touch screen. In combination with a compatible cartridge (containing a screen-printed electrode) and reaction mix, the touch screen interface allows the user to initialize and monitor an electrochemical LAMP reaction in an integrated device.

To test whether this system could more broadly handle amplification of RNA and DNA from different pathogens, LAMPlify was evaluated on several clinically relevant pathogens. SARS-CoV-2, Influenza A and Influenza B were tested using previously published primer sets^14,26,27^ (As1e, and S4 for SARS-CoV-2, IAV for Influenza A, and IBV for Influenza B) with 10^5^ copies of the corresponding synthetic or extracted targets (Figure 2B). A large separation in normalized peak currents was observed for all primer sets within 8 to 10 min after reaction start compared to the respective no template controls.

**Figure 2:**
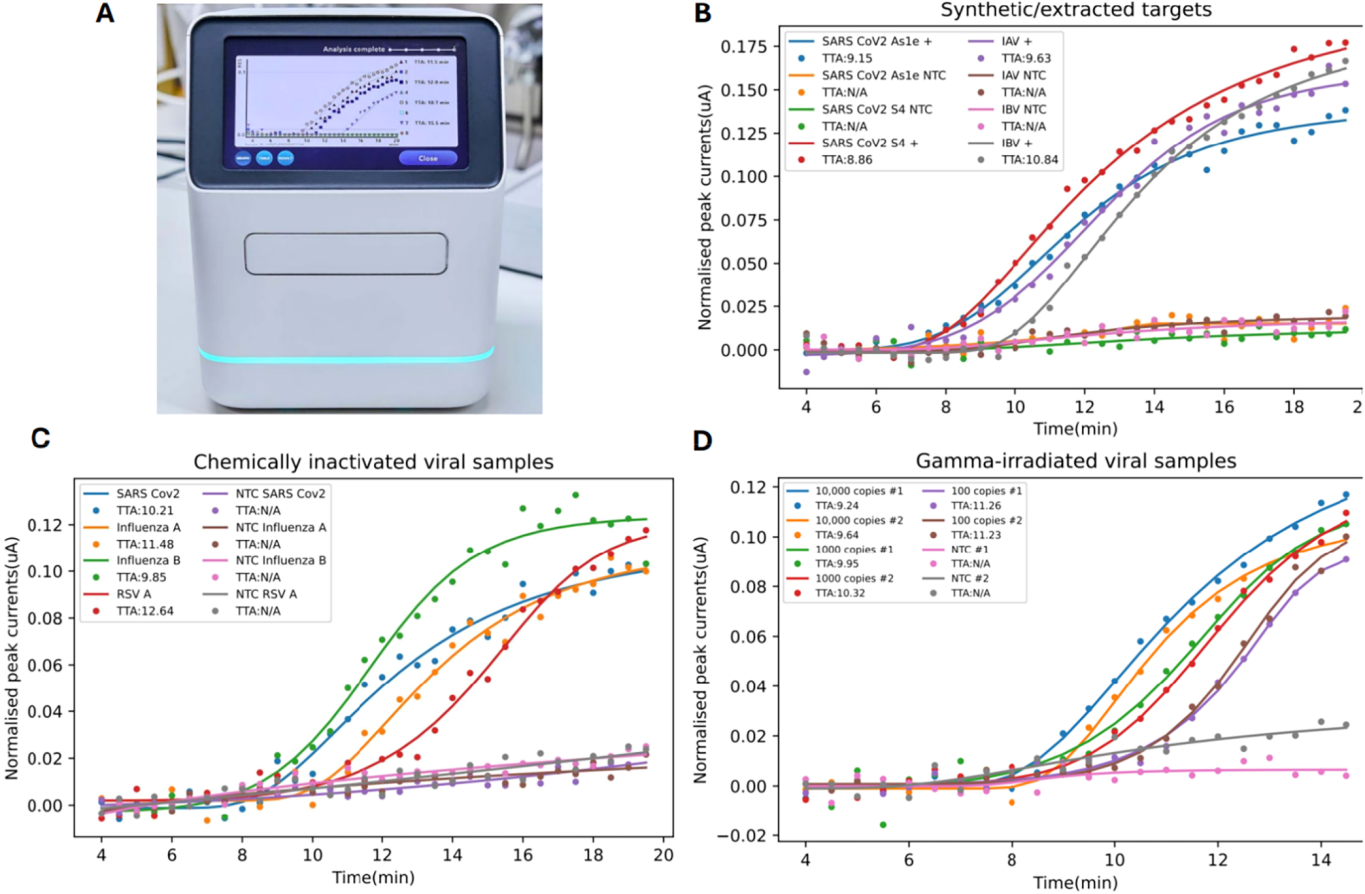
LAMPlify point-of-care instrument enables real-time eLAMP detection of SARS-CoV-2, IAV, IBV, and RSV A using synthetic targets, purified RNA, and inactivated viruses (A) LAMPlify Instrument. (B) Reaction progress of point-of-care LAMP system on synthetic or extracted genomic targets for 10^5^ copies per reaction of SARS CoV-2, IAV, IBV. (C) Reaction progress for purified chemically inactivated viral samples of SARS CoV-2, IAV, IBV, RSV A. (D) Reaction progress for γ-irradiated inactivated viral samples of SARS CoV-2 at copy numbers ranging from 102 to 104. Normalized peak currents (ΔμA)″ are computed as the difference in currents at −0.73 V and −0.85 V, using a 5-point moving average at each potential and baseline subtraction using the lag phase (3–7 min). Panels (B–D) show representative real-time ΔμA traces from individual reactions, illustrating separation between positive samples and corresponding negative controls; sample calling was based on the predefined positivity criteria described in Methods (see Logistic Curve Fitting).

### Inactivated Viral Samples

To demonstrate the assay’s capability on viral particles, chemically inactivated and γ-irradiated viral samples were tested directly without prior RNA extraction or purification steps.

In the case of chemical inactivation, shown in Figure 2C, SARS CoV-2, Influenza A, Influenza B and Respiratory syncytial virus A were evaluated with the As1e, IAV, IBV, RSV A primer-sets, respectively.^14,26,27^ All 4 previously published primer sets show clear separation between positively spiked samples and their respective no template control (NTC) confirming that LAMPlify broadly works on LAMP reactions in general and is not specific to a specific primer set–target combination.

To further simulate clinical conditions, γ-irradiated SARS-CoV-2 viral samples were spiked into nasopharyngeal swab samples from a healthy volunteer and tested using the As1e primer-set at quantified target amounts ranging from 10^4^ to 10^2^ copies, shown in Figure 2D. A delay was observed between reaction start and when the normalized peak currents (a proxy for reaction progress) began to rapidly increase. As the quantified initial target concentration was reduced by factors of ten, this time evolved from 9.24 to 9.64 min to 9.95–10.32 min and finally 11.23–11.26 min. The observed inverse correlation between amplification time and sample concentration suggests potential for quantitative analysis. However, a full characterization of this effect is beyond the scope of this work and left for future study.

### Purified Patient Samples

To assess the method’s viability on patient sourced samples and any genetic diversity present, 234 purified clinical samples were tested for SARS CoV-2, IAV, IBV or RSV A using the primer sets mentioned earlier. For each virus, a set of purified viral RNA from patient samples (30 positive, 30 negative, confirmed by RT-qPCR at time of acquisition and as part of this study) was used as a direct comparison between electrochemical LAMP (eLAMP) technology and the RT-qPCR gold standard (see Table 1). Viral RNA was purified from clinical specimens using the MagaBio Plus Virus RNA Purification Kit II (BSC87, Bioer) on the GenePure Pro Nucleic Acid Purification System (NPA-^32^P, Bioer), according to the manual. Six samples were excluded from the study due to disagreement between RT-qPCR classification at time of acquisition and this study. For 231 of the 234 (98.7%) samples evaluated, we found agreement between the eLAMP and RT-qPCR data. Furthermore, when each of the 3 samples with discrepancies were rerun, agreement was obtained. The correlation between LAMPlify and RT-qPCR is highlighted in Figure S4 in the Supporting Information, with all pathogens displaying significant positive Pearson and Spearman rank correlation coefficients (*p* < 0.05) with strengths ranging from **≈**0.9 in the case of SARS-CoV-2 down to **≈**0.4 in the case of RSV A. Representative real-time traces for purified patient samples (positive vs negative) are shown in Figure 3 (additional examples in Figure S5).

**Figure 3:**
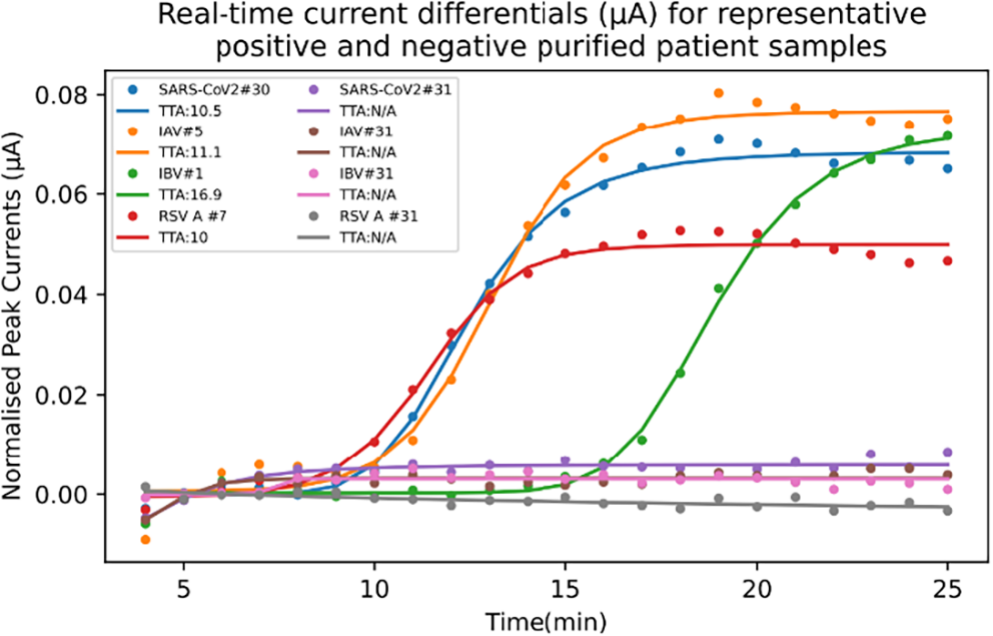
Representative real-time ΔμA traces for one RT-qPCR-positive and one RT-qPCR-negative purified patient sample for SARS-CoV-2, IAV, IBV and RSV A. Each trace corresponds to a single patient sample; quantitative performance metrics for the full cohort (sensitivity, specificity, and overall agreement with RT-qPCR) are reported in Table 1, with individual sample data in Table S3A,B.

**Table 1:** First-Pass Performance of the LAMPlify Assay Compared with RT-qPCR in Detecting Respiratory Viruses in Purified Patient Samples

eLAMP/RT-qPCR	Positive (Sensit. %)	Negative (Specif. %)	Total (Acc. %)
CoV-2	30/30 (88.6–100)	29/30 (83.3–99.4)	59/60 (91.1–99.7)
IAV	27/28 (82.3–99.4)	28/28 (87.9–100)	55/56 (90.6–99.7)
IBV	29/30 (83.3–99.4)	30/30 (88.6–100)	59/60 (91.1–99.7)
RSV	28/28 (87.9–100)	30/30 (88.6–100)	58/58 (93.8–100)
Total	114/116 (93.9–99.5)	117/118 (95.4–99.9)	231/234^a^ (96.3–99.6)

a Table reports first-pass eLAMP vs RT-qPCR agreement (231/234 concordant = 98.7%). The three initially discordant samples were retested by eLAMP, which then matched the RT-qPCR classification (234/234 = 100% after repeat testing).

The observed agreement between LAMPlify and RT-qPCR supports the feasibility of the electrochemical readout in purified clinical RNA under the conditions tested. Because these measurements were performed on extracted RNA rather than direct clinical swabs, they should be interpreted as analytical validation rather than full real-world point-of-care validation.

### Analytical Sensitivity and Quantitative Performance

To determine the Limit of Detection (LOD) and assess the quantitative capabilities of the LAMPlify system, serial dilutions of synthetic Influenza A H1N1 RNA were analyzed. An initial range-finding study (4 replicates per concentration) identified the tentative LOD between 25 and 50 copies per reaction (Figure 4A). This was confirmed through a verification study utilizing 20 replicates at 50 copies/reaction, which resulted in a 100% detection rate (20/20 positive) (Figure 4B). Consequently, the experimentally determined LOD for Influenza A is 50 copies/reaction. Furthermore, the system exhibited potential for semiquantitative analysis. An inverse correlation was observed between the Time to Amplification (TTA) and the log-transformed RNA copy number across a dynamic range of 101 to 104 copies (Figure 4A) with a correlation of *r* = −0.79. While inherently less quantitative than qPCR, these results suggest the device can distinguish between high and low viral loads.

**Figure 4:**
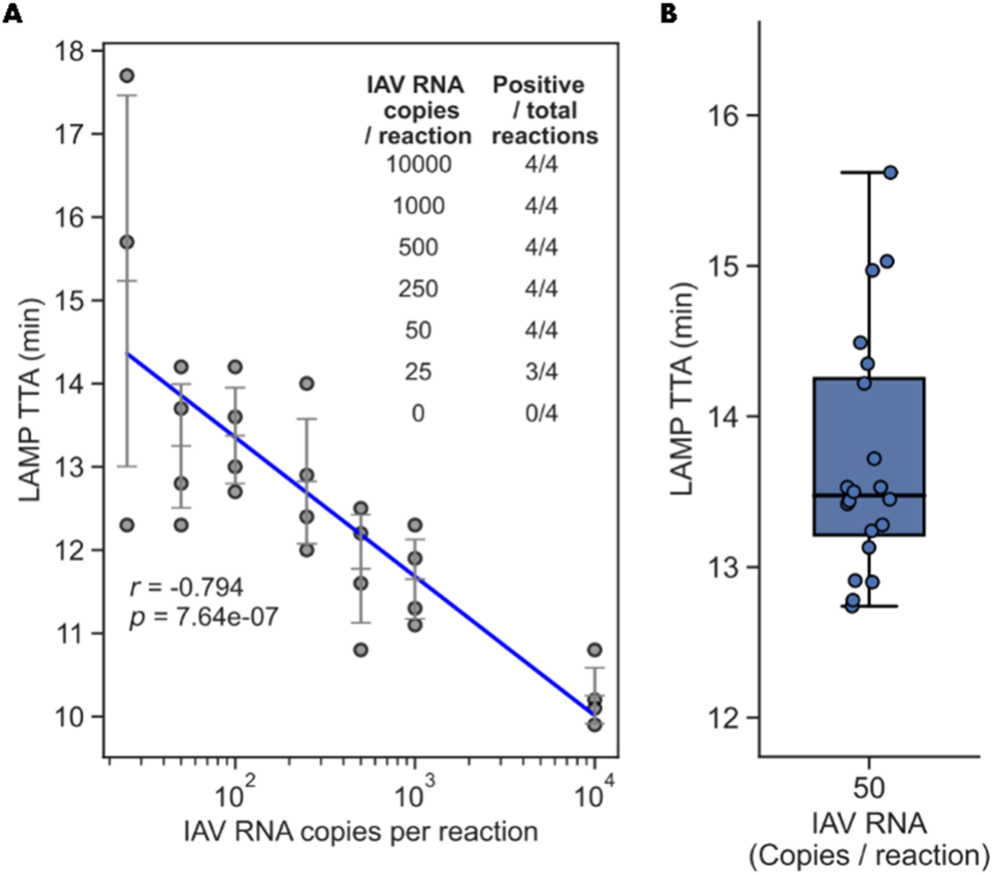
Analytical sensitivity and quantitative performance of the LAMPlify assay using synthetic Influenza A H1N1 RNA. (A) Evaluation of dynamic range and quantitative response. Time to Amplification (TTA) is plotted against the log-transformed RNA copy number per reaction (range 10^1^ to 10^4^ copies). Individual replicates (*n* = 4 per concentration) are shown as open circles, with mean ± SD overlaid at each concentration. The solid line indicates a log–linear regression fit (*r* = −0.79), demonstrating an inverse correlation between viral load and detection time. (B) Determination and verification of the Limit of Detection (LOD). 20/20 replicates of 50 copies per reaction of IAV RNA are detected and the TTA plotted (box plot: median, IQR, range; individual replicates overlaid).

### Extraction-Free Direct-Swab Testing in Equine Samples

A notable feature of LAMP is that, in specific workflows, it can operate without complex nucleic-acid extraction or purification, which is relevant for point-of-care-oriented applications (e.g., direct swab eluates in a low-buffer-capacity lysis buffer). As a proof-of-concept for minimal preanalytical handling within a specific low-buffer lysis workflow, equine nasal swabs were tested with LAMPlify for Equid gammaherpesvirus 5 (EHV-5) and benchmarked against qPCR. Fourteen equine nasal swabs were directly immersed in Diagonal Bio Lysis buffer. For the direct-swab workflow, swabs were immersed in Diagonal Bio lysis buffer and an aliquot was added directly to the LAMP assay (no extraction and no separate prelysis heating). For the qPCR comparison, extraction was first performed using Zymo Research’s Quick-DNA Microprep kit. For the LAMPlify assay, LAMP primers were designed to target the ORF8 (glycoprotein B) gene of the EHV-5 genome using a consensus of 107 sequences obtained from NCBI GenBank. The qPCR assay utilized previously reported primers targeting the E11 gene.^28^

Samples were classified as positive or negative based on whether any amplification was observed before the end of the assay, 20 min in the case of LAMPlify and 40 cycles in the case of qPCR. Both methods made the same positive/negative classification for all 14 samples (9 positive and 5 negative by qPCR). A strip plot of the LAMP time-to-amplification (TTA) of all 14 samples highlighted the agreement between the two assays is presented in Figure 5. A scatter plot of TTA vs qPCR cycle threshold (Ct) is reported in the Supporting Information (Figure S6). This shows that higher Ct values tend to be associated with longer TTA but does not rise to the level of statistical significance, likely due to the limited number of positive samples and the additional variability expected in direct swab/lysis measurements. Nevertheless, the complete agreement observed in this small cohort supports the feasibility of an extraction-free workflow for this specific matrix/analyte combination. Broader claims regarding extraction-free performance across matrices will require larger studies spanning additional pathogens, transport media, and inhibitor-rich sample types. However, because the readout relies on amplification-associated acidification in a lightly buffered system, highly buffered or inhibitor-rich matrices (e.g., blood, soil) may require additional pretreatment and/or dedicated controls.

**Figure 5:**
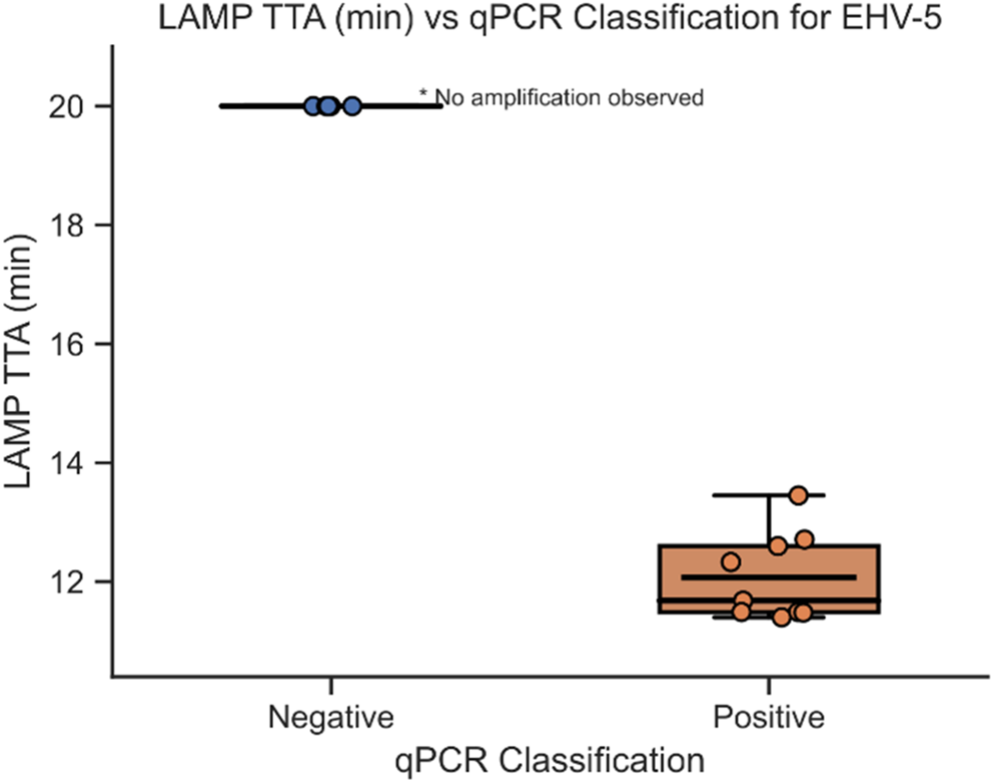
Extraction-free equine nasal swab testing for EHV-5 by LAMPlify shows complete agreement with qPCR. LAMP time-to-amplification (TTA) for 14 individual equine samples classified as positive (*n* = 9) or negative (*n* = 5) for EHV-5 by qPCR. Positive group shown as a box plot (median, IQR, range) with individual samples overlaid. Negative samples did not meet positivity criteria, and their LAMP TTA is plotted at the maximum assay runtime for visualization purposes only.

While these results demonstrate the potential of the LAMPlify system, several limitations must be acknowledged. First, because the assay relies on amplification-associated acidification in a lightly buffered system, performance may depend on sample matrix composition; highly buffered transport media or inhibitor-rich matrices (e.g., standard UTM/VTM, blood, or soil) may require additional pretreatment to avoid false negatives or delayed amplification. Second, the proof-of-concept for direct, extraction-free swab analysis (EHV-5) was conducted on a relatively small sample size (*n* = 14) and should be interpreted as preliminary, necessitating larger clinical cohorts and multiple electrode production lots to confirm robustness. Third, although TTA showed semiquantitative trends, the strength of correlation with RT-qPCR Ct varied by target and was only moderate for some pathogens (e.g., RSV A, with a Spearman rank correlation of approximately 0.4), supporting qualitative (positive/negative) classification and broad viral-load discrimination more strongly than precise quantification.

## Conclusion

In summary, LAMPlify is an integrated electrochemical platform that monitors LAMP amplification in real time through a pH-responsive redox reporter. Under the conditions tested, the system showed strong agreement with RT-qPCR in purified clinical RNA (231/234 concordant samples across SARS-CoV-2, Influenza A, Influenza B, and RSV A). The system also demonstrated feasibility for direct analysis of chemically inactivated and γ-irradiated viral particles without prior RNA extraction, and supported a proof-of-concept extraction-free workflow in equine nasal swabs for EHV-5 (14/14 concordant samples relative to qPCR). These results support the feasibility of voltammetric LAMP for compact diagnostic workflows, while broader validation across prospective clinical cohorts and additional raw sample matrices remains an important next step.

## Supplementary Material



## Notes

Current–voltage (I–V) curves for electrochemically monitored LAMP reactions with Actin Template; reaction progress for Actin LAMP monitored by fluorescence; heat map differential, for varying subtraction potentials; scatter plot of LAMPlify TTA vs RT-qPCR and representative signal traces for purified patient samples for SARS-CoV-2, IAV, IBV, RSV A; scatter plot of LAMPlify TTA vs qPCR Ct for directly analyzed equine samples; colorimetric response of phenol red at various pH; table of LAMP and RT-qPCR primers used; table of Ct values and Time to Positive for purified patient samples (PDF)
