# Supplementary material for: A Novel Voltammetric Point-of-Care Device for Rapid and Accurate Nucleic Acid and Viral Pathogen Detection
Source: ACS Omega. 2026 Jun 1;11(23):33523–31. doi: 10.1021/acsomega.5c12087 (PMC13280907; doi:10.1021/acsomega.5c12087)
Supplement: Supplementary file 1 [file ao5c12087_si_001.pdf]

A novel voltammetric point-of-care device for rapid and accurate nucleic acid and viral pathogen detection.

Roberto Munita‡[1,5,6], Roman Lyttleton‡*[1], Emelie Danefur[1], Sviataslau Sasinovich[4], Karin Wehlin[1], Tautgirdas Ruzgas[2,3]*, Patrik Medstrand[4], Jae Yen Shin[1], Andreas Nyberg[1,8], Kushagr Punyani[1,7]

1. Diagonal Bio AB, The Spark Medicon Village, Scheeletorget 1, Lund, 22381, Sweden
2. Biomedical Science, Faculty of Health and Society, Malmö University, Malmö, 20506, Sweden
3. Biofilms Research Centre for Biointerfaces, Malmö University, Malmö, 20506, Sweden
4. Clinical Virology, Department of Translational Medicine, Lund University, Lund, 22100, Sweden
5. Department of Biochemistry and Molecular Biology, Facultad de Ciencias Químicas y Farmacéuticas, Universidad de Chile, Santiago, 8380494, Chile
6. Advanced Center for Chronic Diseases, Facultad de Ciencias Químicas y Farmacéuticas, Universidad de Chile, Santiago, 1025000, Chile
7. Nested Bio AB, Nannas gata 10, Malmö, 21535, Sweden
8. Nyberg Exploration AB, Regementsgatan 33, Malmö, 21753, Sweden

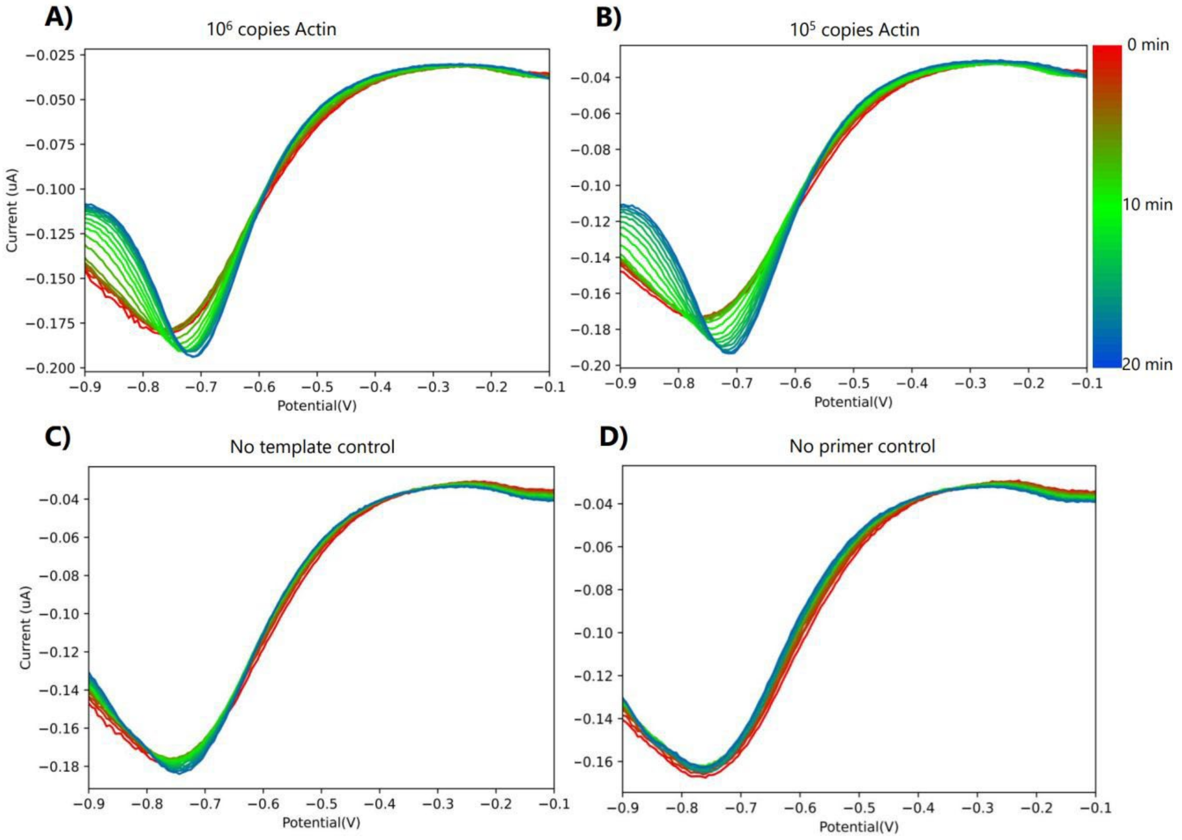

Figure S1. Current-Voltage (I-V) curves for electrochemically monitored LAMP reactions with Actin Template in the following amounts: **A)** 10^6 copies per reaction, **B)** 10^5 copies per reaction, **C)** No template control, **D)** No primers control.

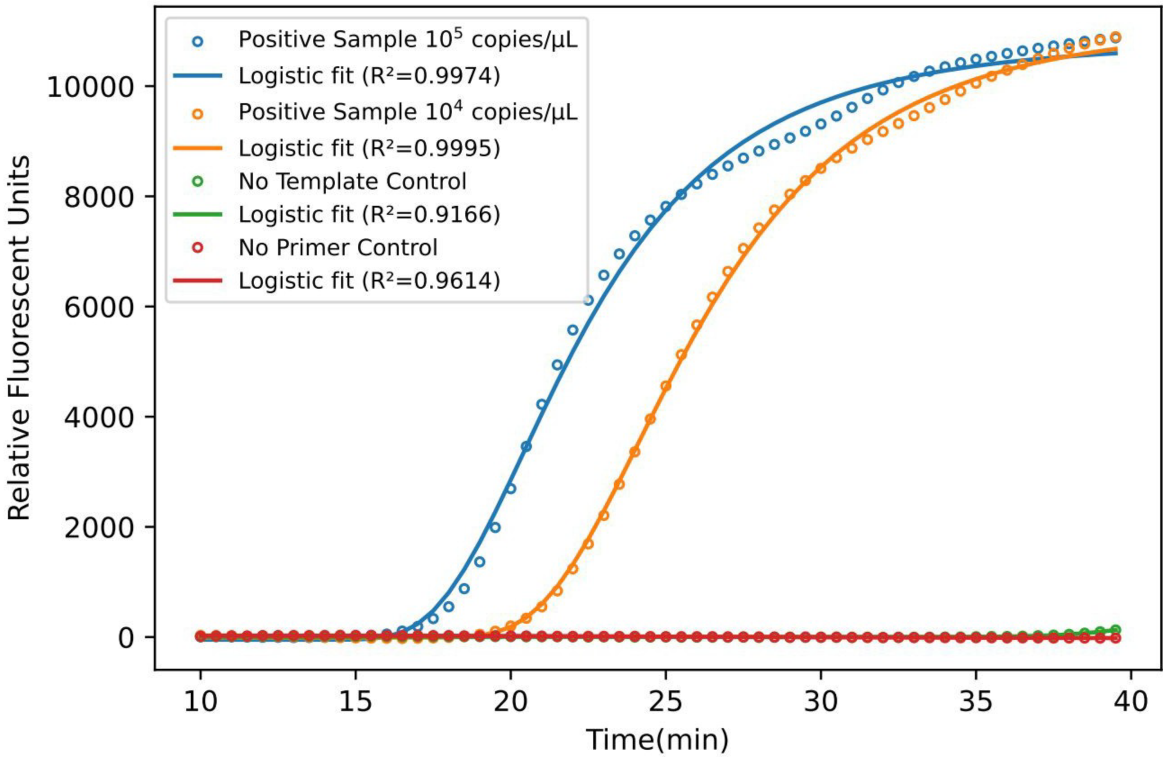

Figure S2. Reaction progress for Actin monitored by fluorescence. LAMP Fluorescent dye B1700s (New England Biolabs) was added to the reaction mix, incubated at 65 °C for 40min using a CFX96 Real-Time PCR Detection System (Bio-Rad). Fluorescence is monitored every 30 seconds.

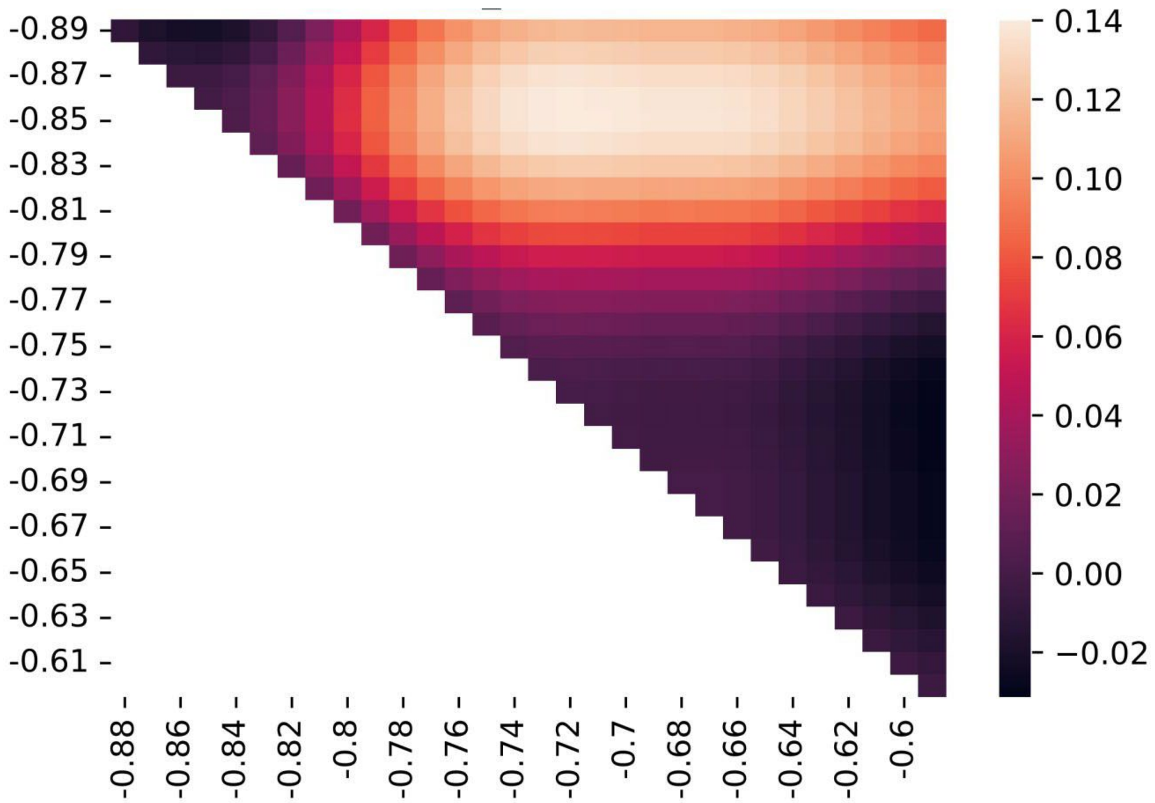

Figure S3. Heat map displaying the difference in saturation currents between positive and negative samples, for varying subtraction potentials.

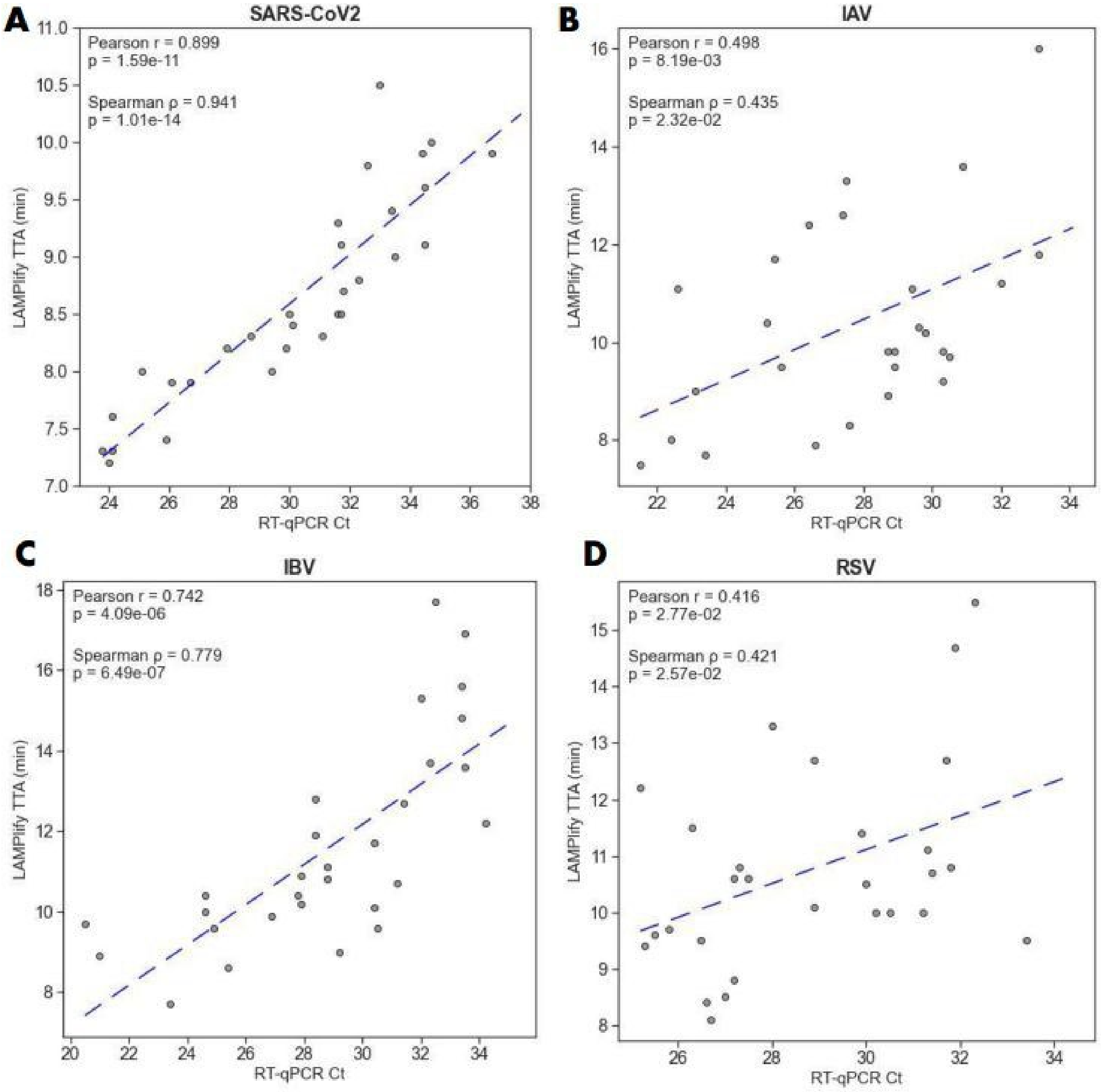

Figure S4. LAMPlify TTA vs RT-qPCR Ct for purified patient samples classified by RT-qPCR as positive for: **A)** SARS-CoV-2, **B)** IAV, **C)** IBV, **D)** RSV A. The Pearson correlation coefficient (**r**), Spearman rank corellation (ρ) and their statistical significance (**p**) were calculated using the pearsonr and spearmanr function of the python package scipy, respectively.

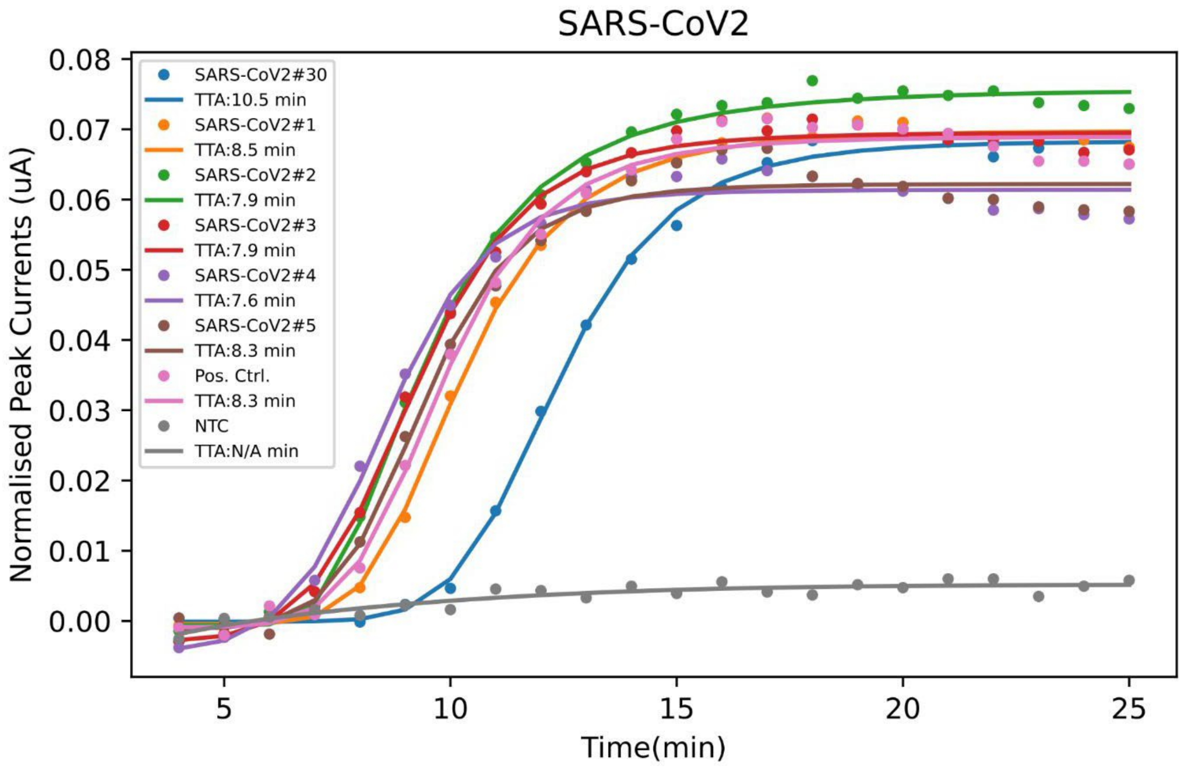

Figure S5A. Representative normalised peak currents (μA) vs time (min) of the purified patient samples for the duration of acquisition for SARS-CoV-2.

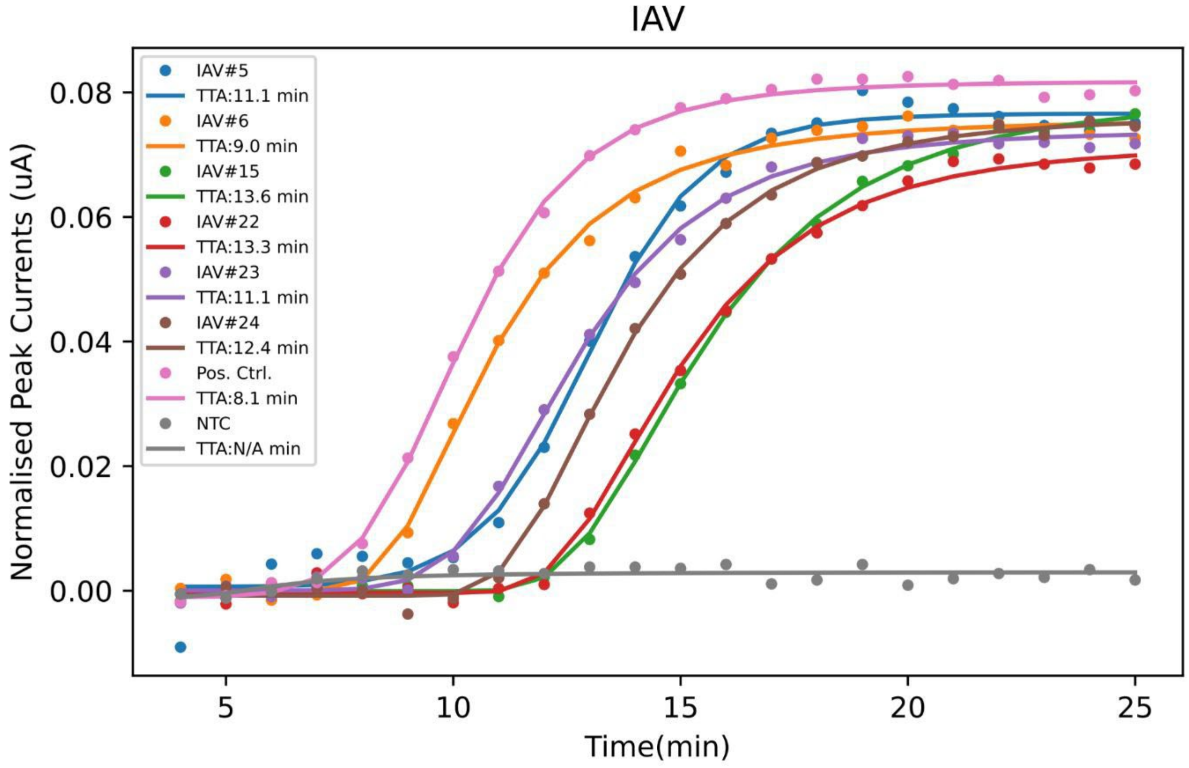

Figure S5B. Representative normalised peak currents (μA) vs time (min) of the purified patient samples for the duration of acquisition for IAV.

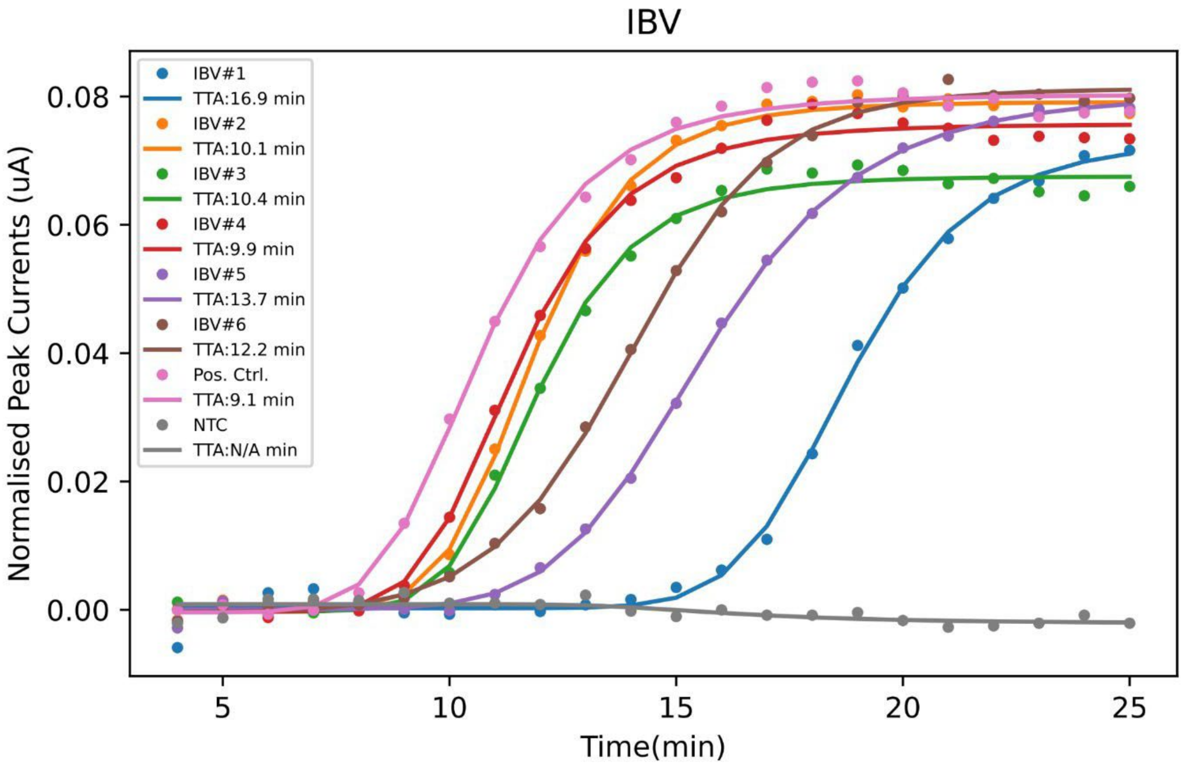

Figure S5C. Representative normalised peak currents (µA) vs time (min) of the purified patient samples for the duration of acquisition for IBV.

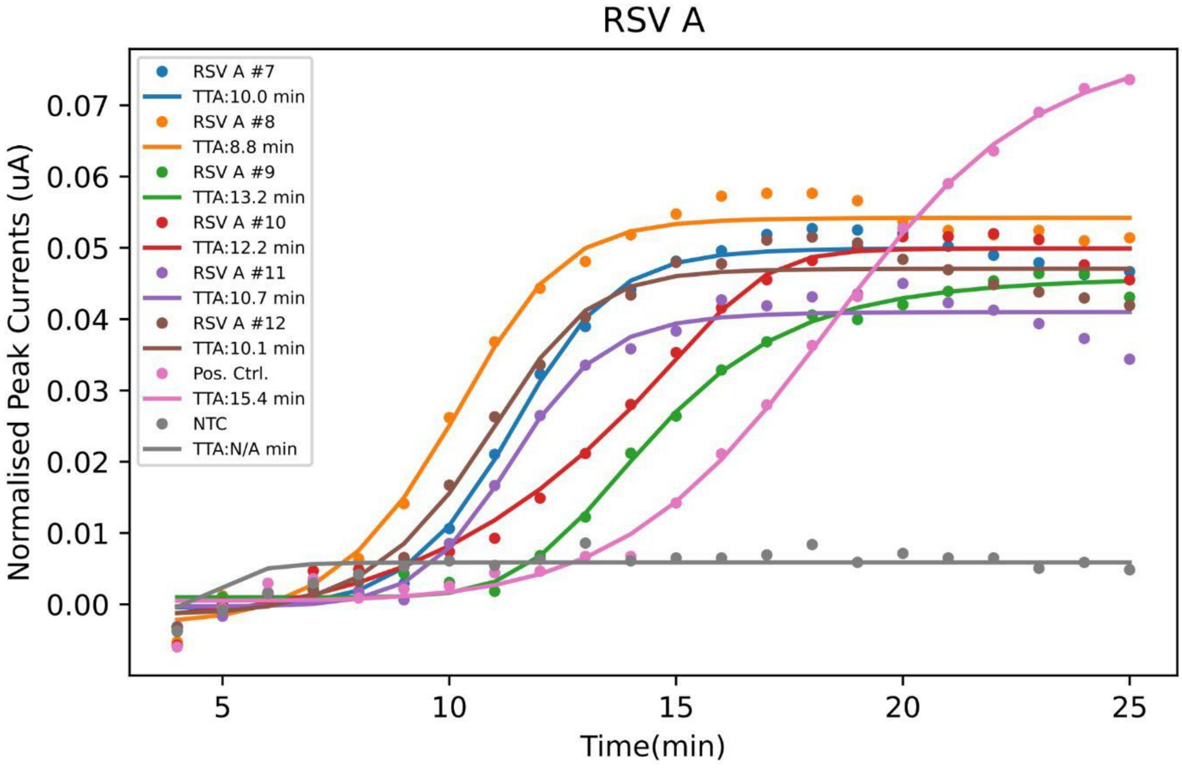

Figure S5D. Representative normalised peak currents (µA) vs time (min) of the purified patient samples for the duration of acquisition for RSV A.

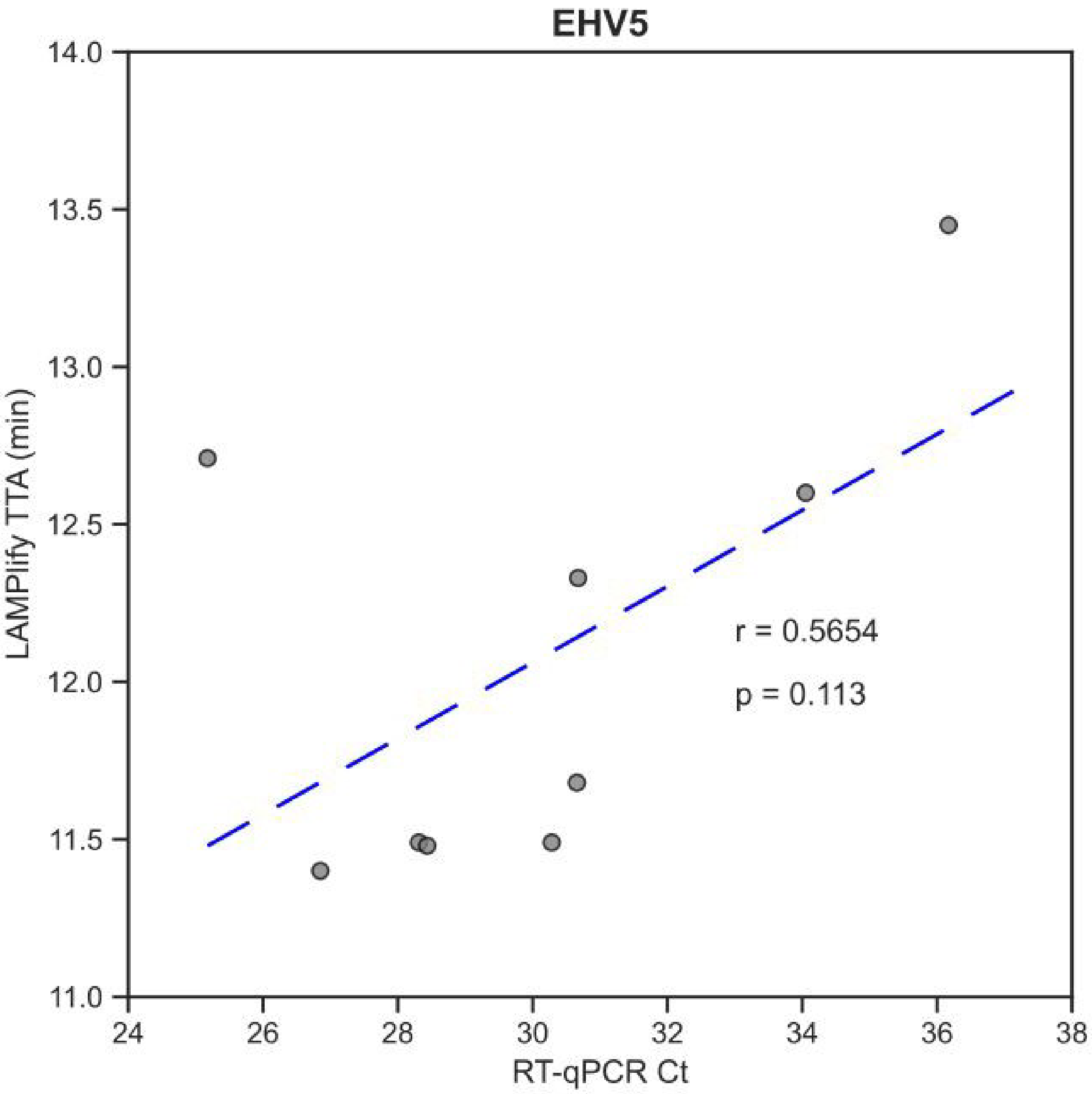

Figure S6. LAMPlify TTA vs RT-qPCR Ct for EHV-5 positive equine samples. The Pearson correlation coefficient (**r**) and its statistical significance (**p**) were calculated using the pearsonr function of the python package scipy.

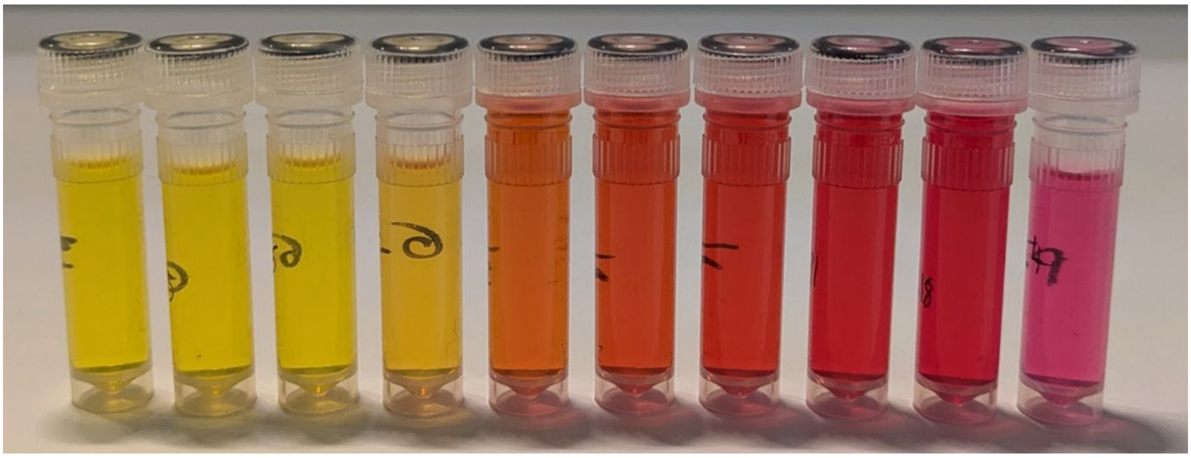

Figure S7. Colorimetric response of phenol red at various pH. pH of solutions (left to right): 5.5, 5.9,6.2,6.4,6.8,7.0,7.3,7.6,7.9,8.4.

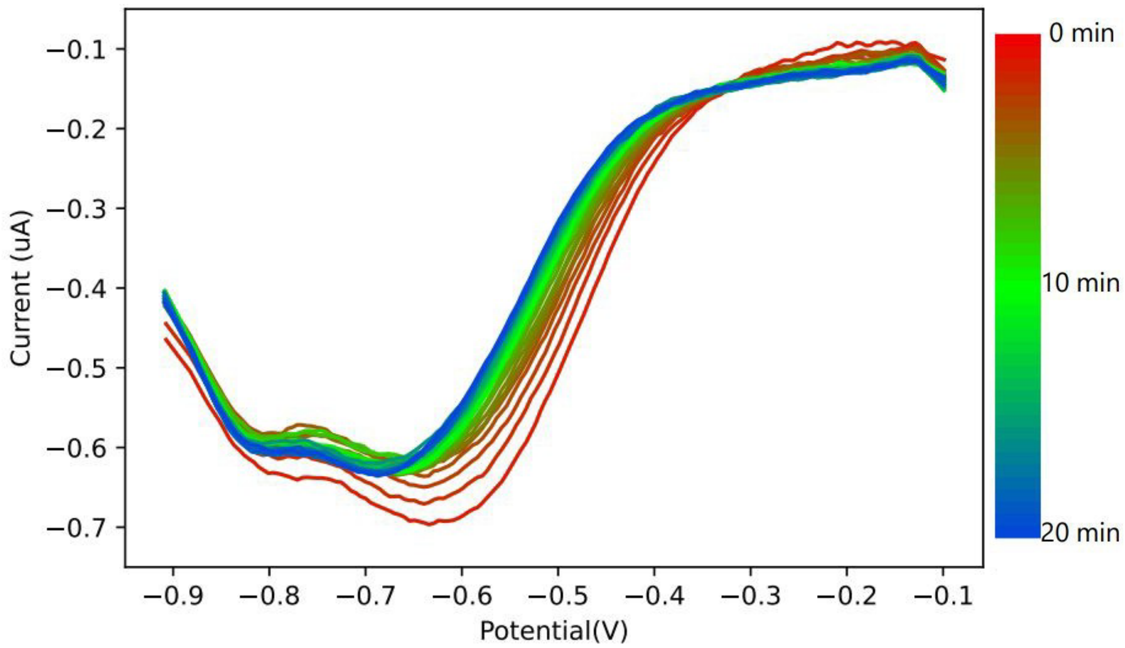

Figure S8A. Representative I-V curve of a negative (as classified by qPCR) extraction free analysis of an equine nasal swab for EHV-5.

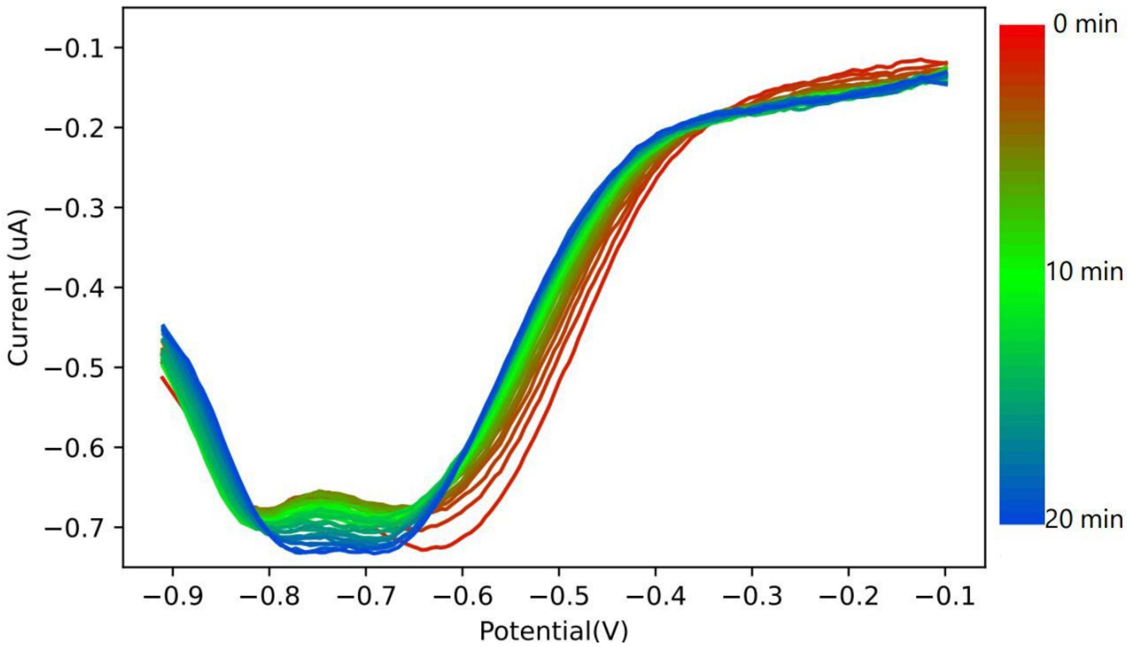

Figure S8B. Representative I-V curve of a positive (as classified by qPCR) extraction free analysis of an equine nasal swab for EHV-5.

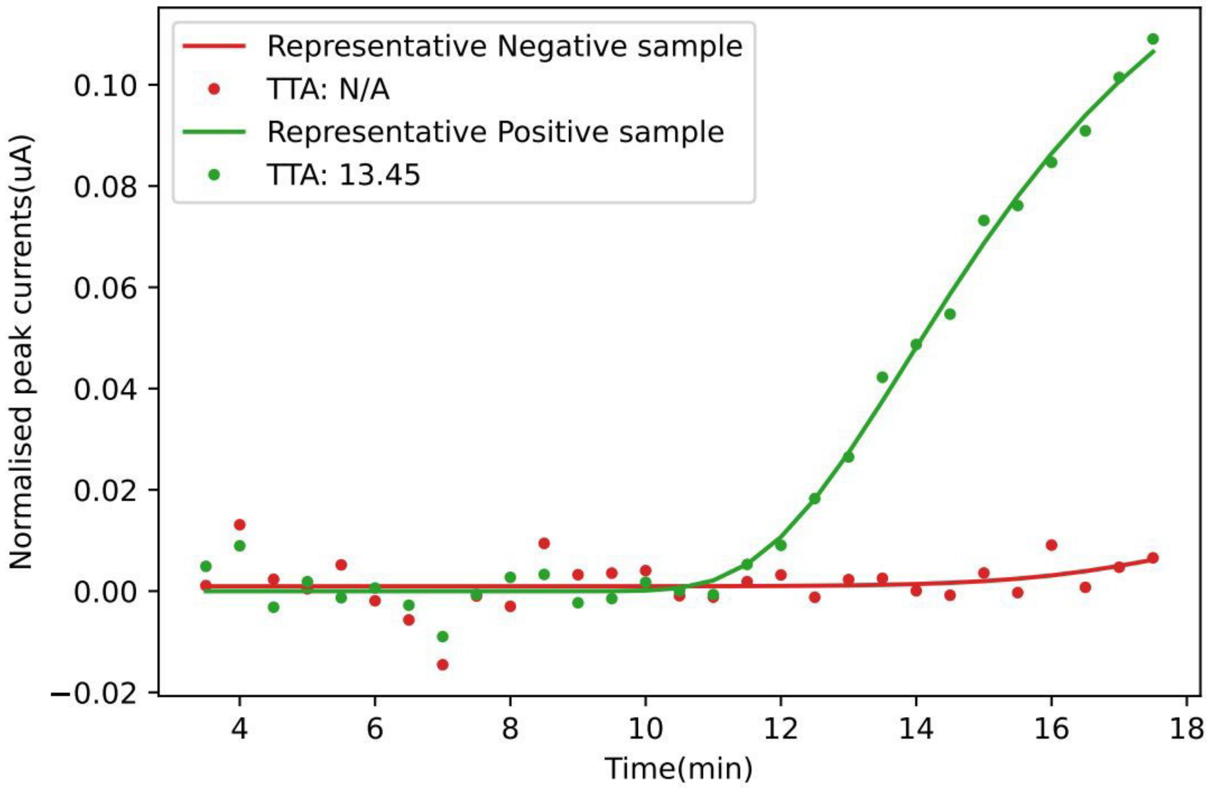

Figure S8C. Normalised peak currents (μA) of the representative negative sample (Fig. S8A) and representative positive sample (Fig. S8B) plotted alongside each-other for the duration of the reaction.

Table S1. LAMP Primers

Target	Primer	Sequence	Source
SARS-CoV2	F3	CGGTGGACAAATTGTCAC	Zhang et. al (2022) [21]
	B3	CTTCTCTGGATTTAACACACTT	
	LF	TTACAAGCTTAAAGAATGTCTGAACACT	
	LB	TTGAATTTAGGTGAAACATTTGTCACG	
	FIP	TCAGCACACAAAGCCAAAAATTTATTTTTCTGTGCAAAGGAAATTAAGGAG	
	BIP	TATTGGTGGAGCTAAACTTAAAGCCTTTTCTGTACAATCCCTTTGAGTG	
IAV	F3-1*	GACTTGAAGATGTCTTTGC	Zhang et. al (2021) [22]
	F3-2*	GACTGGAAAGTGTCTTTGC	
	B3-1*	TRTTATTTGGGTCTCCATT	
	B3-2*	TRTTGTTTGGGTCCCCATT	
	FIP	TTAGTCAGAGGTGACARRATTGCAGATCTTGAGGCTCTC	
	BIP	TTGTKTTCACGCTCACCGTGTTTGGACAAAGCGTCTACG	
	LF	GTCTTGTCTTTAGCCA	
	LB	CMAGTGAGCGAGGACTG	
IBV	F3	GCAACCAATGCCACCATA	Zhang et. al (2021) [22]
	B3	TTCTCTCTTCAAGRGACATC	
	FIP	TAGTCAAGGGCYCTTTGCCACTTTGAAGCAGGAATTCTGGA	
	BIP	CAAGACCGCCTAAACAGACTAAACTTTTACTTTCAGGCTCACTT	

	LF	TGAAAGYCTTTCATAGCAC	
	LB	CAAGAATAAAGACTCACAAC	
RSV A	F3	GAGTTGAAGGGATTTTTGCA	Takayama et. al (2019) [23]
	B3	TGGGTTGTTCAATATATGGTAGA	
	FIP	TAACTGATTTTGCTAAGACCCCCGGATTGTTTATGAAT GCCTATGG	
	BIP	CAAGCAGAAATGGAACAAGTTGTGCTGCTTCTCCACC CAATT	
	LF	CACCGTAACATCACTTG	
	LB	GAGGTGTATGAGTATGCTCAGA	
EHV 5	-	Undisclosed (commercially available)	EHV5 test kit (Diagonal Bio)
*Equal copies of similar primers are combined to create coverage of a common SNP in the target genetic marker.			

Table S2. RT-qPCR primers

Target	Primer	Sequence	Source
SARS-CoV2	FW	CTGCAGATTTGGATGATTTCTCC	Shu et. al (2021) [15]
	RV	CCTTGTGTGGTCTGCATGAGTTTAG	
IAV	FW-1*	CAAGACCAATCYTGTCACCTCTGAC	Shu et. al (2021) [15]
	FW-2*	CAAGACCAATYCTGTCACCTYTGAC	
	RV-1*	GCATTYTGGACAAAVCGTCTACG	
	RV-2*	GCATTTTGGATAAAGCGTCTACG	
IBV	FW	TCCTCAAYTCACTCTTCGAGCG	Shu et. al (2021) [15]
	RV	CGGTGCTCTTGACCAAATTGG	
RSV A	FW	AAGTCTTTGTGGTGGAACCTGTCT	Todd et. al (2019) [16]
	RV	CGCCCCTGCTAAAATCCATA	
EHV 5	FW	AAGTCTTTGTGGTGGAACCTGTCT	Zarski et. al (2017) [23]
	RV	CGCCCCTGCTAAAATCCATA	
*Equal copies of similar primers are combined to create coverage of a common SNP in the target genetic marker.			

Table S3A – Positive Purified Patient Samples

		RT-qPCR (Sample taken)	RT-qPCR (This Study)	eLAMP (This Study)		
Sample Number	Grou p	Ct	Ct	Classification	TTA min	Comments
1	IAV	27.1	ND	Positive	16.2	RT-qPCR disagreement. Excluded.
2	IAV	21	28.7	Positive	9.8	
3	IAV	27.7	37.0	Negative	ND	False Negative. Re-test positive 13,2 min
4	IAV	21.2	32.0	Positive	11.2	
5	IAV	23.3	29.4	Positive	11.1	
6	IAV	18.1	23.1	Positive	9.0	
7	IAV	26.4	ND	Positive	20.0	RT-qPCR disagreement. Excluded.
8	IAV	23.1	30.3	Positive	9.8	
9	IAV	22.7	22.4	Positive	8.0	
10	IAV	24.4	28.7	Positive	8.9	
11	IAV	23.5	26.6	Positive	7.9	
12	IAV	23.4	29.6	Positive	10.3	
13	IAV	19.7	29.8	Positive	10.2	
14	IAV	29.1	23.4	Positive	7.7	
15	IAV	23.6	30.9	Positive	13.6	
16	IAV	23.6	30.5	Positive	9.7	
17	IAV	17.8	30.3	Positive	9.2	
18	IAV	18.6	28.9	Positive	9.8	
19	IAV	22.5	28.9	Positive	9.5	
20	IAV	16.4	21.5	Positive	7.5	
21	IAV	17.5	27.6	Positive	8.3	
22	IAV	21.1	27.5	Positive	13.3	
23	IAV	16.3	22.6	Positive	11.1	
24	IAV	18.6	26.4	Positive	12.4	

25	IAV	17	25.2	Positive	10.4	
26	IAV	17.1	25.4	Positive	11.7	
27	IAV	19.5	27.4	Positive	12.6	
28	IAV	23	33.1	Positive	11.8	
29	IAV	15	25.6	Positive	9.5	
30	IAV	24.2	33.1	Positive	16.0	
1	IBV	27.3	33.5	Positive	16.9	
2	IBV	24.7	30.4	Positive	10.1	
3	IBV	22.2	27.8	Positive	10.4	
4	IBV	18	26.9	Positive	9.9	
5	IBV	24.1	32.3	Positive	13.7	
6	IBV	26.2	34.2	Positive	12.2	
7	IBV	27	33.5	Positive	13.6	
8	IBV	16.7	25.4	Positive	8.6	
9	IBV	24.1	31.2	Positive	10.7	
10	IBV	15.7	29.0	Negative	ND	False Negative. Re-test positive 9,4 min
11	IBV	21.8	29.2	Positive	9.0	
12	IBV	19.8	27.9	Positive	10.9	
13	IBV	18.8	28.8	Positive	10.8	
14	IBV	24.7	33.4	Positive	15.6	
15	IBV	22.5	28.4	Positive	12.8	
16	IBV	12.2	32.5	Positive	17.7	
17	IBV	24.9	31.4	Positive	12.7	
18	IBV	21.8	27.9	Positive	10.2	
19	IBV	19.7	21.0	Positive	8.9	
20	IBV	20.1	24.6	Positive	10.0	
21	IBV	17.1	20.5	Positive	9.7	
22	IBV	19.4	23.4	Positive	7.7	
23	IBV	23	24.9	Positive	9.6	
24	IBV	21	24.6	Positive	10.4	
25	IBV	26.8	30.4	Positive	11.7	
26	IBV	26.4	30.5	Positive	9.6	

27	IBV	25	33.4	Positive	14.8	
28	IBV	27.5	32.0	Positive	15.3	
29	IBV	21.1	28.4	Positive	11.9	
30	IBV	21.6	28.8	Positive	11.1	
1	CoV2	22.8	30.0	Positive	8.5	
2	CoV2	19.4	26.7	Positive	7.9	
3	CoV2	16.6	26.1	Positive	7.9	
4	CoV2	16.5	24.1	Positive	7.6	
5	CoV2	23.7	28.7	Positive	8.3	
6	CoV2	25.4	31.8	Positive	8.7	
7	CoV2	25.5	32.3	Positive	8.8	
8	CoV2	22.7	25.1	Positive	8.0	
9	CoV2	15.3	24.1	Positive	7.3	
10	CoV2	16.2	23.8	Positive	7.3	
11	CoV2	23.3	31.6	Positive	9.3	
12	CoV2	27.4	32.6	Positive	9.8	
13	CoV2	17.7	24.0	Positive	7.2	
14	CoV2	23.3	29.9	Positive	8.2	
15	CoV2	25.7	31.7	Positive	9.1	
16	CoV2	21.4	29.4	Positive	8.0	
17	CoV2	21.9	27.9	Positive	8.2	
18	CoV2	24.2	31.1	Positive	8.3	
19	CoV2	28.1	34.5	Positive	9.6	
20	CoV2	25.4	33.4	Positive	9.4	
21	CoV2	23.4	30.1	Positive	8.4	
22	CoV2	26.3	36.7	Positive	9.9	
23	CoV2	22.9	31.7	Positive	8.5	
24	CoV2	26.5	33.5	Positive	9.0	
25	CoV2	28.1	34.4	Positive	9.9	
26	CoV2	25.8	34.5	Positive	9.1	
27	CoV2	27.1	34.7	Positive	10.0	
28	CoV2	15.8	25.9	Positive	7.4	
29	CoV2	21.1	31.6	Positive	8.5	

30	CoV2	25.7	33.0	Positive	10.5	
1	RSV A	21.6	26.5	Positive	9.5	
2	RSV A	24.1	32.3	Positive	15.5	
3	RSV A	19.7	28.9	Positive	12.7	
4	RSV A	20.3	27.3	Positive	10.8	
5	RSV A	21.2	31.7	Positive	12.7	
6	RSV A	23.5	27.5	Positive	10.6	
7	RSV A	25.4	30.5	Positive	10.0	
8	RSV A	24.5	ND	Positive	8.8	RT-qPCR disagreement. Excluded.
9	RSV A	22.2	28.0	Positive	13.3	
10	RSV A	19.8	25.2	Positive	12.2	
11	RSV A	24	31.4	Positive	10.7	
12	RSV A	20.6	28.9	Positive	10.1	
13	RSV A	21.5	26.7	Positive	8.1	
14	RSV A	20.8	33.4	Positive	9.5	
15	RSV A	22.3	29.9	Positive	11.4	
16	RSV A	22.5	26.6	Positive	8.4	
17	RSV A	23.2	30.2	Positive	10.0	
18	RSV A	18.2	25.5	Positive	9.6	
19	RSV A	19.5	26.3	Positive	11.5	

20	RSV A	24.3	27.0	Positive	8.5	
21	RSV A	23.2	31.2	Positive	10.0	
22	RSV A	20.7	27.2	Positive	8.8	
23	RSV A	21.2	ND	Negative	ND	RT-qPCR disagreement. Excluded.
24	RSV A	22.6	31.3	Positive	11.1	
25	RSV A	16.7	25.3	Positive	9.4	
26	RSV A	22	31.8	Positive	10.8	
27	RSV A	20.3	27.2	Positive	10.6	
28	RSV A	23.9	30.0	Positive	10.5	
29	RSV A	23.7	31.9	Positive	14.7	
30	RSV A	18.5	25.8	Positive	9.7	

Table S3B – Negative Purified Patient Samples

	RT-qPCR				eLAMP (TTA min)				
Sample Number	Ct CoV2	Ct IAV	Ct IBV	Ct RSV	TTA CoV2	TTA IAV	TTA IBV	TTA RSV	Comments
31	ND	ND	ND	ND	ND	ND	ND	ND	
32	ND	37.4	ND	ND	ND	13.8	ND	ND	RT-qPCR IAV disagreement. IAV Excluded.
33	ND	ND	ND	ND	ND	ND	ND	ND	
34	0.0	38.9	0.0	0.0	0.0	0.0	0.0	0.0	RT-qPCR IAV disagreement. IAV Excluded.
35	ND	ND	ND	ND	ND	ND	ND	ND	

36	ND	ND	ND	ND	ND	ND	ND	ND	
37	ND	ND	ND	ND	ND	ND	ND	ND	
38	ND	ND	ND	ND	ND	ND	ND	ND	
39	ND	ND	ND	ND	ND	ND	ND	ND	
40	ND	ND	ND	ND	ND	ND	ND	ND	
41	ND	ND	ND	ND	ND	ND	ND	ND	
42	ND	ND	ND	ND	ND	ND	ND	ND	
43	ND	ND	ND	ND	ND	ND	ND	ND	
44	ND	ND	ND	ND	ND	ND	ND	ND	
45	ND	ND	ND	ND	18.7	ND	ND	ND	False Positive eLAMP CoV2. The retest was negative
46	ND	ND	ND	ND	ND	ND	ND	ND	
47	ND	ND	ND	ND	ND	ND	ND	ND	
48	ND	ND	ND	ND	ND	ND	ND	ND	
49	ND	ND	ND	ND	ND	ND	ND	ND	
50	ND	ND	ND	ND	ND	ND	ND	ND	
51	ND	ND	ND	ND	ND	ND	ND	ND	
52	ND	ND	ND	ND	ND	ND	ND	ND	
53	ND	ND	ND	ND	ND	ND	ND	ND	
54	ND	ND	ND	ND	ND	ND	ND	ND	
55	ND	ND	ND	ND	ND	ND	ND	ND	
56	ND	ND	ND	ND	ND	ND	ND	ND	
57	ND	ND	ND	ND	ND	ND	ND	ND	
58	ND	ND	ND	ND	ND	ND	ND	ND	
59	ND	ND	ND	ND	ND	ND	ND	ND	
60	ND	ND	ND	ND	ND	ND	ND	ND	
